# Detection of Dengue Virus in *Aedes aegypti* Eggs Supports Vertical Transmission in Natural Settings

**DOI:** 10.3390/microorganisms14081810

**Published:** 2026-08-17

**Authors:** Tiago Souza Salles, Brenda Martins Vasconcellos, Carlucio Rocha-Santos, Maria Luiza Maia, Svetoslav Nanev Slavov, Renata Campos Azevedo, Lucio Ayres Caldas, Monica Ferreira Moreira

**Affiliations:** 1 Instituto de Química, Universidade Federal do Rio de Janeiro, Rio de Janeiro 21941-909, RJ, Brazil; tiagosouzasalles@gmail.com (T.S.S.); brendamartinsvasc@gmail.com (B.M.V.); maria_.maia@hotmail.com (M.L.M.); 2 Laboratório de Sinalização Celular Programa de Biologia Molecular e Biotecnologia, Instituto de Bioquímica Médica Leopoldo de Meis, Universidade Federal do Rio de Janeiro, Rio de Janeiro 21941-909, RJ, Brazil; carluciorsantos@gmail.com; 3 Laboratório de Biologia, Controle e Vigilância de Insetos Vetores, Fundação Oswaldo Cruz, Rio de Janeiro 21040-900, RJ, Brazil; 4 Center for Viral Surveillance and Serological Assessment (CeVIVAs), Butantan Institute, São Paulo 05503-900, SP, Brazil; svetoslav.slavov@fundacaobutantan.org.br; 5 Departamento de Virologia, Instituto de Microbiologia Paulo de Góes, Universidade Federal do Rio de Janeiro, Av. Carlos Chagas Filho, 373, Rio de Janeiro 21941-970, RJ, Brazil; renatacampos@micro.ufrj.br; 6 Laboratorio de Ultraestrutura Celular Hertha Meyer, Instituto de Biofísica Carlos Chagas Filho, Centro de Pesquisas em Medicina de Precisao, Universidade Federal do Rio de Janeiro, Rio de Janeiro 21941-902, RJ, Brazil; lucio@biof.ufrj.br; 7 Núcleo Multidisciplinar de Pesquisas em Biologia—NUMPEX-BIO, Campus Duque de Caxias Geraldo Cidade, Universidade Federal do Rio de Janeiro, Duque de Caxias, Rio de Janeiro 25245-390, RJ, Brazil

**Keywords:** *Aedes aegypti*, eggs, dengue virus (DENV), vertical transmission, epidemiological surveillance, transovarial transmission

## Abstract

Dengue remains one of the most important mosquito-borne viral diseases worldwide, and vector control continues to be the cornerstone of disease prevention. Vertical transmission has been proposed as a mechanism contributing to the maintenance of dengue virus (DENV) during interepidemic periods. In this study, field-collected egg pools and second-generation eggs derived from field-collected females from the state of Rio de Janeiro were analyzed by RT-qPCR, Sanger sequencing, plaque assay, and transmission electron microscopy. The second-generation egg samples (E1–E4), corresponding to F2 eggs of the AEDES-RIO strain, were all DENV positive. Sample E1 was positive for DENV-1, whereas samples E2–E4 were positive for DENV-4, confirming transovarial vertical transmission. The detection of DENV-1 and DENV-2 in field-collected egg pools further supports the occurrence of vertical transmission. All PCR-amplified viral fragments were confirmed by Sanger sequencing. Furthermore, homogenized egg samples naturally infected with DENV successfully infected Vero cells, demonstrating that the virus remained infectious. Transmission electron microscopy revealed virus-like particles of approximately 50 nm in diameter within egg tissues, providing ultrastructural evidence that corroborated the molecular and virological findings. Collectively, these findings provide additional evidence supporting vertical transmission of DENV in naturally infected *Aedes aegypti* populations and highlight the potential of egg-based surveillance as a complementary tool for entomological monitoring. However, further longitudinal studies are needed to establish its epidemiological value.

## 1. Introduction

Dengue is one of the most important mosquito-borne viral diseases worldwide and remains a major public health concern in tropical and subtropical regions [1,2]. Despite the availability of specific dengue vaccines they cannot interrupt transmission and therefore, vector control remains the most effective strategy for reducing arboviral dissemination [3]. Dengue virus (DENV), the causing agent, belongs to the family *Flaviviridae* (genus *Orthoflavivirus*), and the *Orthoflavivirus denguei* species, according to the most recent International Committee on Taxonomy of Viruses (ICTV) classification [4]. The virus comprises four antigenically distinct serotypes (DENV-1 to DENV-4), all transmitted primarily by *Aedes* mosquitoes [5,6,7,8]. The arbovirus control in Brazil relies mainly on integrated vector management, which includes the elimination of artificial breeding sites and use of insecticides. However, the populations of *Aedes* spp. have developed resistance to these compounds, driven by genetic factors and their widespread and indiscriminate application, reducing significantly the effectiveness of these interventions [9,10]. This is reflected by the extensive DENV epidemics in Brazil, as illustrated by those occurring between 2011 and 2017 that resulted in more than 9 million confirmed dengue cases were reported in the region of the Americas [8]. Only in 2015, there were 2 million cases, an epidemic that caused worldwide concern [7].

Dengue fever exhibits a wide clinical spectrum. Approximately 70% of infections are asymptomatic or subclinical, whereas symptomatic cases range from mild febrile illness to severe outcomes such as dengue hemorrhagic fever (DHF) and dengue shock syndrome (DSS) [7,8]. This broad spectrum in the clinical presentation complicates early detection and highlights the need for complementary surveillance tools beyond human case reporting.

The early DENV detection in mosquito vectors plays an important role in arbovirus surveillance [11]. Identifying infected vectors may help public health authorities to identify areas with viral circulation and strengthen integrated vector management strategies. Complementary entomological surveillance approaches are particularly relevant in regions where insecticide resistance and large number of asymptomatic human infections may limit traditional surveillance strategies. Moreover, the natural reservoir of DENV remains unknown, reinforcing the importance of entomological surveillance. Notably, the detection of the virus in male mosquitoes provides evidence supporting that DENV can be transmitted transovarially [12]. This transmission route may help to explain the occurrence of extensive outbreaks even in the absence of significant human mobility, as newly emerged mosquitoes may already be infected and capable of sustaining viral circulation [13,14,15].

Vertical transmission, including transovarial transmission, has been described for several arboviruses and has been proposed as a mechanism contributing to viral maintenance during interepidemic periods [16,17,18,19,20]. While transovarial transmission specifically refers to viral infection of developing oocytes before oviposition, the broader concept of vertical transmission also encompasses other mechanisms by which viruses may be transmitted from infected females to their offspring. Therefore, the detection of DENV in mosquito eggs provides evidence consistent with vertical transmission but does not, by itself, distinguish the underlying mechanism of viral transfer [17,18,21]. Vertical transmission is particularly relevant under unfavorable environmental conditions for mosquito activity, where it likely contributes to viral persistence when horizontal transmission is reduced or interrupted [19,21]. Evidence of vertical transmission has been reported in both flaviviruses and alphaviruses [18], including Zika virus (ZIKV) in *A. aegypti* [20]. Natural vertical transmission of DENV has been documented in Brazil for more than three decades in both *Aedes aegypti* and *Aedes albopictus*. Previous studies have reported the detection of DENV in eggs, larvae, pupae, and adult mosquitoes from several Brazilian states, including Amazonas, Ceará, Bahia, Mato Grosso, Rio Grande do Norte, São Paulo, Pernambuco, Minas Gerais, and Rio de Janeiro [22,23,24,25,26,27,28,29,30]. Collectively, these studies support the hypothesis that vertical transmission contributes to the maintenance of DENV during interepidemic periods while highlighting the broad geographic distribution of this phenomenon in Brazil.

In the case of DENV, this transmission route has been associated with outbreaks in areas experiencing epidemiological remission [21,31] and is supported by field evidence of DENV detection in larvae and adult male mosquitoes [23,32,33,34,35,36]. Collectively, these findings indicate that vertical transmission contributes to the maintenance of DENV in mosquito populations during interepidemic periods [37] and may influence epidemic dynamics alongside climatic factors and host population immunity [38].

Accordingly, monitoring vertical transmission particularly through the detection of DENV in immature mosquito stages may enhance entomological surveillance by identifying areas at increased risk of outbreaks and serving as a complementary surveillance strategy for impending epidemics, thereby supporting the timely implementation of pre-epidemic interventions.

Therefore, the objective of this study was to directly detect DENV in naturally collected *A. aegypti* eggs using an integrated framework of molecular, virological, and ultrastructural approaches. To this end, field-collected egg pools and F2 eggs derived from field-collected females were analyzed by RT-qPCR, conventional PCR, Sanger sequencing, plaque-forming assays, and transmission electron microscopy. In addition, eggs from mosquitoes experimentally infected with DENV-3 were included to complement the field investigations and to assess DENV transmission under controlled laboratory conditions. Finally, the infectivity of DENV-positive egg homogenates was evaluated in Vero cells to determine whether infectious virus could be recovered from mosquito eggs.

## 2. Materials and Methods

### 2.1. Virus Propagation and Preparation

In this study, DENV-1-4, Zika virus (ZIKV), and Yellow fever virus (YFV) were propagated in African green monkey kidney epithelial (Vero) cells using the same culture protocol. Viruses were inoculated at a multiplicity of infection (MOI) of 0.02 into Vero cells initially grown in $25\;{\mathrm{cm}}^{2}$ tissue culture flasks and maintained at 37 °C in a humidified atmosphere containing 5% ${\mathrm{CO}}_{2}$ in Dulbecco’s Modified Eagle Medium (DMEM; Invitrogen, Waltham, MA, USA) supplemented with 2.5% fetal bovine serum (Gibco-Invitrogen, Waltham, MA, USA), 100 U/mL penicillin, and 100 μg/mL streptomycin (Gibco-Invitrogen). Virus stocks were subsequently expanded in 750 cm^2^ roller bottles and incubated for 7–10 days, or until approximately 50% cytopathic effect (CPE) was observed.

Cell culture supernatants were harvested, clarified, and concentrated using Amicon Ultra-15 centrifugal filters (10,000 NMWL; Millipore, Merck, Burlington, MA, USA), following the protocol described by Putnak et al. [39] with minor modifications, as ultracentrifugation was not performed. Concentrated virus stocks were aliquoted and stored at −80 °C until use. Viral stocks were routinely titrated by plaque-forming assay (PFA) as part of laboratory quality control. However, within the scope of the present study, only the DENV-3 stock was used for the experimental oral infection of *A. aegypti* mosquitoes. The remaining virus stocks (DENV-1, DENV-2, DENV-4, ZIKV, and YFV) were used exclusively as positive controls for the serotype-specific RT-qPCR assays.

### 2.2. Laboratory Mosquito Infection with DENV-3

To evaluate our artificial glass membrane feeding system [40], approximately 200 female *A. aegypti* mosquitoes were offered an infectious blood meal consisting of a 1:1 mixture of defibrinated rabbit blood and a DENV-3 suspension (8 × $10^{7}$ PFU/mL). Mock-infected mosquitoes were fed with a 1:1 mixture of defibrinated rabbit blood and DMEM in the absence of virus. Oral infection experiments were conducted in a dedicated insectary at the Cell Signaling Laboratory, Molecular Biology and Biotechnology Program, Institute of Medical Biochemistry Leopoldo de Meis, Federal University of Rio de Janeiro (IBqM-UFRJ, Rio de Janeiro, Brazil), which is physically separate from the laboratory at the Institute of Chemistry (IQ-UFRJ) where the field-collected egg samples were processed. Based on the estimated blood meal volume, each female ingested approximately 1.4 × $10^{2}$ PFU of DENV-3.

Mosquitoes were allowed to feed for 1 h, and subsequerntly fully engorged females were selected and maintained under controlled insectary conditions (28 °C, 80% relative humidity, and a 12 h light:12 h dark photoperiod) [40]. On the sixth day post-infection, a filter paper strip was placed inside a 30-mL oviposition cup to allow egg laying. After a 24-h oviposition period (day 7 post-infection), the eggs were collected for subsequent analyses.

Following oviposition, the infected females were grouped into three pools of 20 mosquitoes (MA1) for molecular analysis, whereas the eggs were grouped into three pools of 100 eggs (EA3) for downstream molecular and virological analyses. Corresponding mock controls consisted of three pools of 20 mock-infected females (MA2) and three pools of 100 eggs obtained from mock-infected females (EA4), which were processed in parallel throughout all experimental procedures.

### 2.3. Field Egg Collection and Viral RNA Extraction

Field-collected *Aedes* eggs were obtained using standard ovitraps consisting of black plastic containers filled with clean water and fitted with an Eucathex paddle as the oviposition substrate. Ovitraps were deployed at different collection sites throughout the city of Rio de Janeiro and remained in the field for several days to allow natural oviposition by *Aedes* mosquitoes. Egg pools analyzed in this study were obtained through collaborations with partner research groups and public health surveillance programs. The collections were originally performed for independent entomological studies, and representative oviposition paddles were kindly provided for the present proof-of-concept study. Egg samples E1–E4 consisted of F2 eggs of the AEDES-RIO strain, established from field-collected females from the state of Rio de Janeiro and provided by the Avoid Project/FAPERJ. Field-collected egg pools designated UR1–UR4 and PQT1–PQT2 were provided by the LMTH/FIOCRUZ-RJ group, whereas samples CP1–CP3 were provided by LACEN-RJ.

After the collection period, the Eucathex paddles containing mosquito eggs were transported to the laboratory. Approximately 100–300 eggs, depending on the number recovered from each ovitrap, were carefully removed from the paddles using sterile forceps and sterile pipette tips and transferred to 1.5 mL microcentrifuge tubes containing 200 μL of cell culture medium.

**Figure 1:**
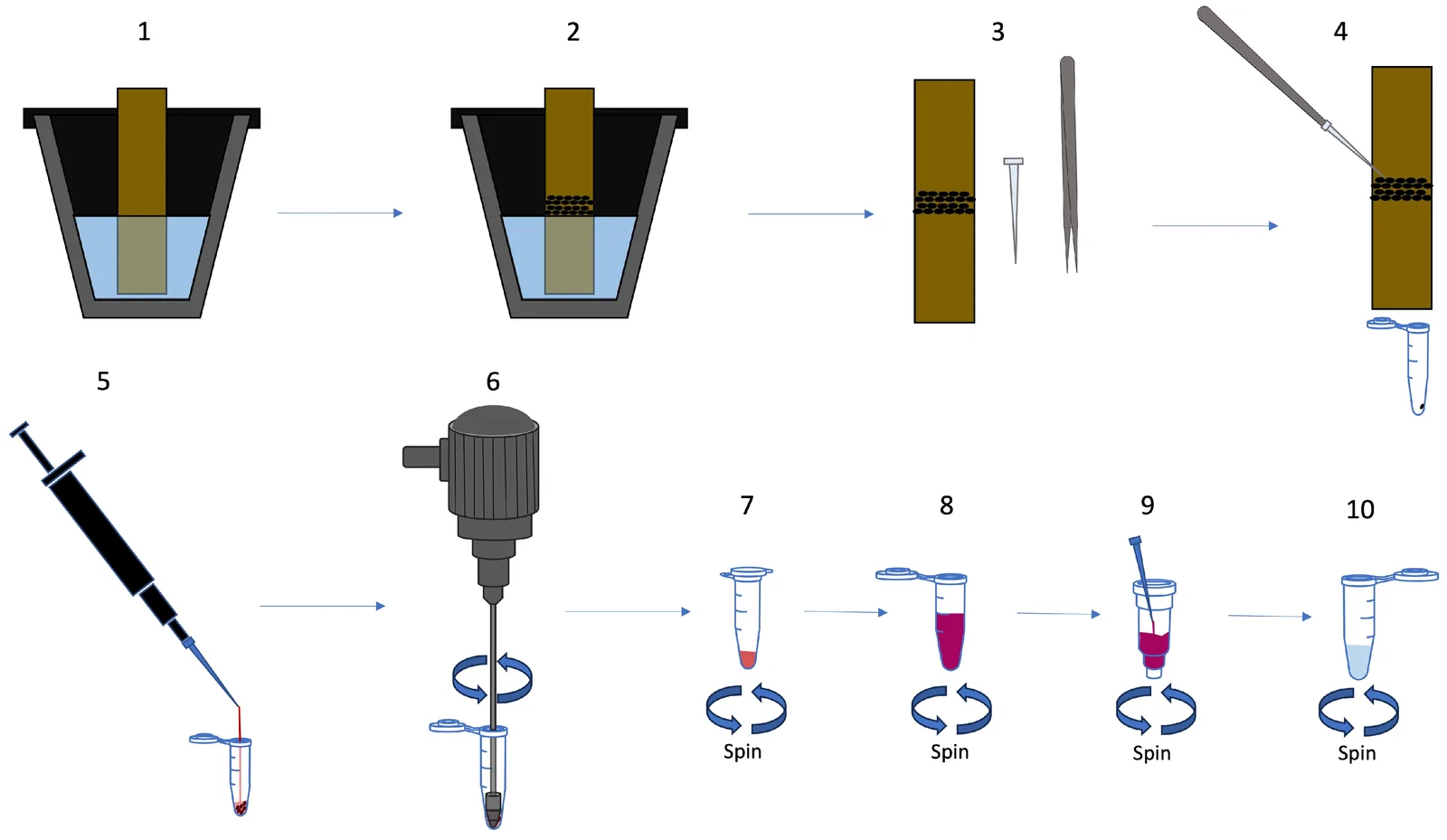
Scheme for collecting and extracting genetic material from *A. aegypti* eggs. (**1**) A small black container (ovitrap) containing clean water and a Eucathex paddle was prepared and placed in the field to attract *Aedes* females for oviposition. (**2**) After a few days, the Eucathex paddle was retrieved (**3**) Tweezers (forceps) and sterile pipette tips were prepared for egg handling. (**4**) Eggs adhered to the paddle were carefully removed using forceps and a sterile tip and transfer-red into a 1.5 mL microtube. (**5**) A total of 200 μL of culture medium was added. (**6**) Mechanical maceration was performed using a rapid-freezing pad. (**7**) The homogenate was centrifuged to remove debris, and 100 μL of the supernatant was transferred to a new microtube (**8**) TRIzol reagent was added according to the Directzol™ Plus kit protocol (**9**) The sample was transferred to a purification column and washed according to the manufacturer’s instructions. (**10**) RNA was eluted into a new microtube for subsequent detection by RT-qPCR.

The egg suspensions were mechanically triturated by repeated rapid freezing in liquid nitrogen followed by disruption using a stainless-steel electric pestle. This freeze–trituration cycle was repeated three times to ensure efficient disruption of the egg chorion and release of intracellular contents. The resulting egg suspensions were centrifuged at 8000× *g* for 10 min to remove debris. The clarified supernatants obtained from all field-collected egg samples were stored at −80 °C until processing for molecular and virological analyses, which were performed contemporaneously with the collection period (2013–2018). One hundred μL of the clarified supernatant was transferred to a new microcentrifuge tube for RNA extraction using the Direct-zol RNA extraction kit according to the manufacturer’s instructions. The remaining supernatant was stored at −80 °C for subsequent virus isolation in Vero cell culture. Purified RNA was subsequently analyzed by RT-qPCR for the detection and serotyping of DENV, as described below. The complete experimental workflow is summarized in Figure 1.

### 2.4. DNase Treatment and cDNA Synthesis

Purified RNA obtained from field-collected egg pools and viral positive controls was treated with DNase I (Fermentas, Burlington, ON, Canada) according to the manufacturer’s instructions. Briefly, 8 μL of RNA was incubated with 1 μL of DNase I buffer prior to reverse transcription. First-strand cDNA synthesis was performed using the High-Capacity cDNA Reverse Transcription Kit (Applied Biosystems, Foster City, CA, USA) according to the manufacturer’s instructions.

### 2.5. Real-Time PCR (qPCR)

For qPCR amplification, the viral cDNA obtained for each serotype and primer sets were used is separate reactions for the identification of DENV serotypes according to the protocol described by Salles et al. [41]. The amplification was performed using Power SYBR Green Mix (Applied Biosystems, Foster City, CA, USA) was used following the reaction conditions and post-amplification analysis recommended by the manufacturer. Reactions had final volumes of 15 μL and contained 5 μL of cDNA at various dilutions, 2.5 μL of each primer set at 400 nM concentration, and 7.5 μL of reaction mix. Analyzes of the melting temperature (Tm) curves included in the qPCR used the following steps: a denaturation at 94 °C for 1 m followed by 78.5 °C for 10 s and a ramp to 94 °C at a rate of 0.1 °C/10 s with continuous fluorescence measurement.

### 2.6. DENV Sequencing

The amplified DENV products from eggs and females mosquitoes were submitted to Sanger sequencing. The PCR amplicon was initially purified and then sequenced by ABI PRISM BigDye Terminator Cycle Sequencing Ready Reaction kit in ABI PRISM DNA Analyzer 3730 equipment (Applied Biosystems, USA) at the PDTIS/FIOCRUZ DNA Sequencing Platform [42]. The obtained sequences were submitted to the Basic Local Alignment Search Tool (BLAST)–NCBI–NIH program (https://www.ncbi.nlm.nih.gov/geo/query/blast.html, accessed on 11 May 2026) for similarity identification.

### 2.7. Transmission Electron Microscopy

The UR1 field-collected *A. aegypti* egg sample, previously identified as positive for DENV by RT-qPCR and confirmed by Sanger sequencing, was selected as a representative sample for ultrastructural analysis. Eggs obtained from the uninfected laboratory *A. aegypti* Rockefeller colony were processed in parallel as negative controls. Eggs were separated and triturated and the first fixation was performed with 2.5% glutaraldehyde in 0.1 M sodium cacodylate buffer (containing 5 mM calcium chloride) for 2 h at room temperature or 24 h at 4 °C. After fixation, the samples were washed three times for one hour with 0.2 M sodium cacodylate buffer solution (pH 7.2) and 0.4% sucrose for 1 h. In the second fixation, the eggs were treated with 1% osmic acid in 0.1M sodium cacodylate buffer for 30 min, at room temperature and protected from light. Subsequently, the sample was dehydrated in successive ethanol baths at concentrations of 50%, 70%, 80%, 90% and twice 100%. The samples were then soaked in EPON resin (EPON 812 polybed), containing DDSA (dodecenyl succinic anhydride), MNA (methyl nadic anhydride) and a catalyst (DMP-30 2,4,6 tridimethyl) and subjected to ultrafine cutting. As described by Reynolds [43], sections are counterstained with uranyl acetate for 30 min and lead citrate for 1 (one) min. The material is then examined under a transmission electron microscope.

### 2.8. Plaque Forming Assay (PFA)

To evaluate whether DENV-positive field-collected *A. aegypti* eggs contained infectious virus, clarified homogenates prepared from selected RT-qPCR-positive egg pools (PQT and E4) were inoculated onto confluent Vero cell monolayers. Vero cells were maintained in DMEM supplemented with 10% fetal bovine serum and antibiotics at 37 °C in a humidified atmosphere containing 5% ${\mathrm{CO}}_{2}$, according to the modified protocol of Volkmer and Rebello [44]. Prior to inoculation, monolayers were washed with PBS, and 700 μL of each egg homogenate or its corresponding fold-10 dilution (-1, -2, -3 and -4) was added to each well. Following a 1-h adsorption period at 37 °C, the inoculum was removed and replaced with an overlay consisting of a 1:1 mixture of 2× DMEM and 4% carboxymethylcellulose (CMC) supplemented with 5% fetal bovine serum. Plates were incubated for 7 days, fixed with 8% formaldehyde overnight, and stained with 1% crystal violet. Plaques were counted, and infectious virus titers were expressed as plaque-forming units per milliliter (PFU/mL).

### 2.9. Data Analysis

Because the present study was designed as a descriptive proof-of-concept investigation, no inferential statistical analyses were performed. Descriptive analyses and graphical data normalization were conducted using GraphPad Prism (version 8; GraphPad Software, San Diego, CA, USA). No inferential statistical analyses were performed because the objective of the study was to characterize DENV detection in field-collected *A. aegypti* egg samples rather than to compare experimental groups. Results are presented as mean Ct values, estimated viral copy numbers, detection frequencies, minimum infection rates (MIR), and plaque-forming units (PFU/mL), as appropriate.

## 3. Results

### 3.1. DENV Detection in Field-Collected Egg Pools

A total of 13 field-collected egg pools, comprising 3050 *A. aegypti* eggs, obtained from different locations in the Rio de Janeiro State were analyzed for the presence of DENV by RT-qPCR. DENV RNA was detected in 10 of the 13 pools (76.9%), including samples collected from the Metropolitan Region of Rio de Janeiro, Urca, Paquetá Island, Bangu, and Vila Kennedy (Table 1). The geographic distribution of the sampling sites is shown in Supplementary Figure S1. DENV RNA was detected in 10 of the 13 analyzed egg pools, representing three DENV serotypes (DENV-1, DENV-2, and DENV-4), whereas three pools (UR3, UR4, and PQT2) were negative for all investigated DENV serotypes. The distribution of DENV serotypes among the analyzed egg pools is summarized in Table 1. Information regarding the number of pools analyzed, pool sizes, and minimum infection rates (MIR) is presented in Table 2, while quantitative RT-qPCR data, including Ct values and estimated viral genome copy numbers, are provided in Supplementary Table S1.

DENV-1 was the most frequently detected serotype, being identified in egg pools collected of F2 generation of the AEDES-RIO strain, Urca, Ilha de Paquetá, Vila Kennedy and Bangu. DENV-2 was detected in pools from Ilha de Paquetá Vila Kennedy and Bangu, whereas DENV-4 was detected exclusively in samples collected from Ilha do Governador (Table 1).

### 3.2. Laboratory Infection with DENV-3

Approximately 200 female *A. aegypti* mosquitoes were orally infected with DENV-3 through an infectious blood meal. Based on the estimated blood meal volume, each female ingested approximately 1.4 × 10^2^ PFU of virus. Fully engorged females were maintained under controlled laboratory conditions until oviposition. Three biological pools of infected females (MA1) and three corresponding pools of eggs (EA3) were analyzed by RT-qPCR together with their respective mock-infected controls (MA2 and EA4). Samples were grouped into pools, with approximately 300 eggs per pool, and subjected to the detection of DENV serotypes 1–4 by RT-qPCR and conventional PCR [41]. Among the analyzed groups, DENV-3 was detected in both MA1 (orally infected mosquitoes) and EA3 (their corresponding egg samples), and its presence was subsequently confirmed by Sanger sequencing. DENV-3 RNA was detected exclusively in MA1 and EA3, whereas all mock control pools remained negative. Representative Ct values are presented in Supplementary Table S1. Conventional nested PCR followed by Sanger sequencing confirmed the identity of the amplified DENV-3 fragments.

### 3.3. Detection of DENV in Rio de Janeiro and Metropolitan Region (2013–2016)

Representative DENV-positive egg pools identified by RT-qPCR were further analyzed by conventional nested PCR followed by Sanger sequencing. Sequence analysis confirmed the identity of DENV-1, DENV-2, and DENV-4 detected in the corresponding field-collected samples (Supplementary Figure S1). The F2 egg pools of the AEDES-RIO strain were donated by the members of the Avoiding Bites Project/FAPERJ [45]. RT-qPCR analysis [41] performed on the eggs of the F2 generation detected DENV-1 in group E1 and DENV-4 in groups E2–E4. Additionally, DENV-4 was confirmed by Sanger sequencing of the amplified fragments. The UR1, UR2, and PQT sample sets were provided by LMTH/FIOCRUZ-RJ, whereas the CP1, CP2, and CP3 sample sets were collected in the Bangu and Vila Kennedy neighborhoods of Rio de Janeiro in 2016. All analyzed samples were positive for DENV-1, while the PQT and CP1–CP3 samples showed co-infection with DENV-1 and DENV-2. The nucleotide sequences and their corresponding BLAST analyses confirming DENV serotype identity are provided in Supplementary File S1. In contrast, no DENV RNA was detected in the UR3, UR4, and PQT2 egg pools (Table 1 and Table 2).

**Figure 2:**
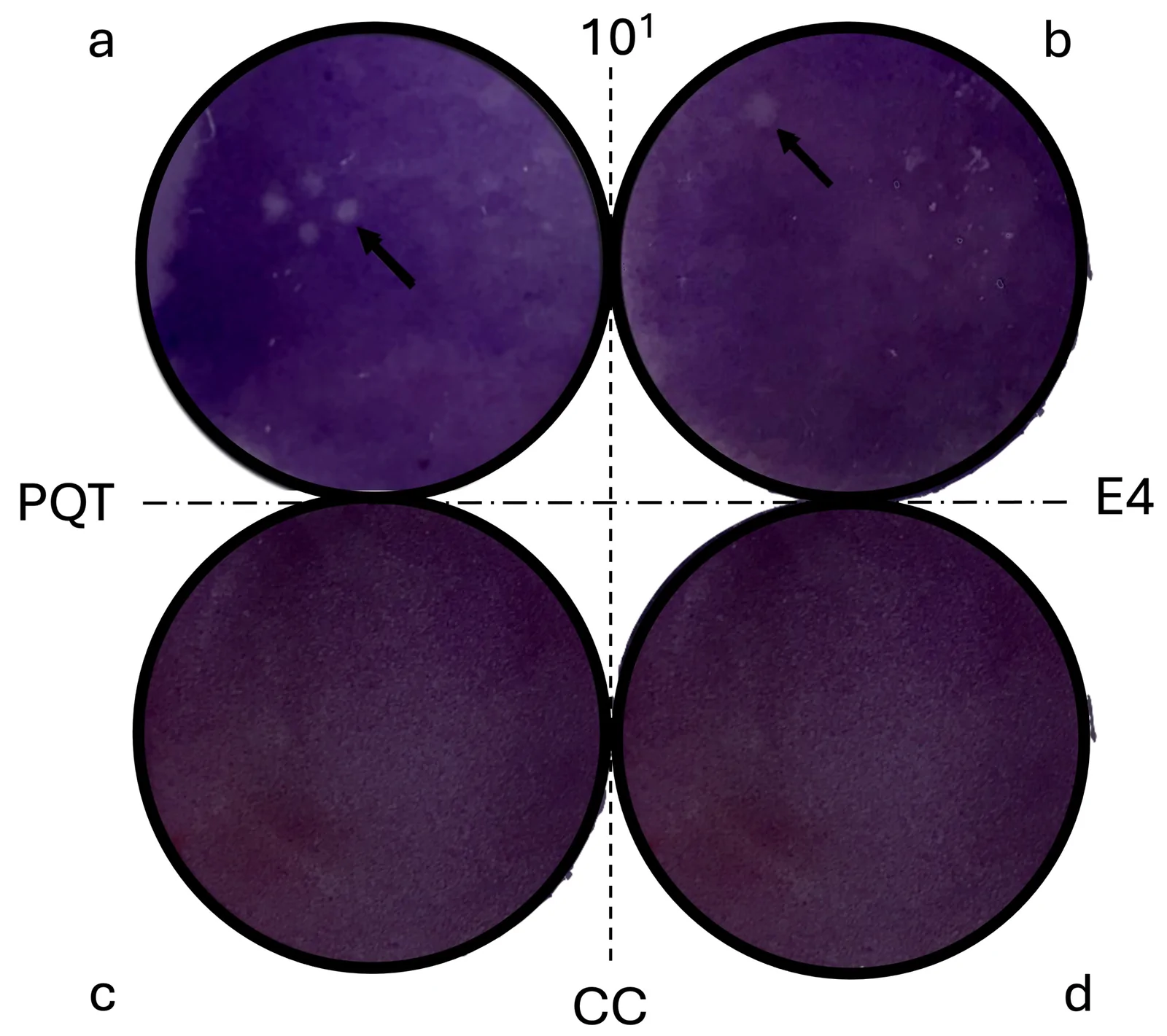
Plaque forming assay (PFA) of the PQT and E4 samples, with cell lysis in infected Vero cells, indicated by the arrows. Infectivity of macerated egg samples (PQT and E4) in Vero cells. Macerated egg samples (PQT and E4) were used to infect confluent Vero cell monolayers: (**a**) represents a well of the 6-well plate equivalent to a $10^{1}$ dilution of sample PQT, (**b**) represents a well of the 6-well plate equivalent to a $10^{1}$ dilution of sample E4, (**c**) represents a well of the 6-well plate equivalent to the assay cell control (CC) of sample PQT (CC—Cell Control), and (**d**) represents a well of the 6-well plate equivalent to the assay cell control (CC) of sample E4.

To determine whether DENV detected in mosquito eggs remained infectious, clarified homogenates prepared from two RT-qPCR-positive egg pools (PQT1 and E4) were inoculated onto confluent Vero cell monolayers. Cytopathic plaques were observed seven days after infection, demonstrating the recovery of infectious virus from both samples (Figure 2). The PQT1 homogenate yielded four plaques at the $10^{-1}$ dilution, corresponding to an estimated infectious titer of 5.7 × $10^{2}$ PFU/mL, whereas the E4 homogenate yielded one plaque at the same dilution, corresponding to 1.4 × $10^{2}$ PFU/mL.

These findings demonstrate that infectious DENV was present in both egg samples. Because E4 consists of F2 eggs derived from field-collected females, it provides evidence consistent with transovarial vertical transmission. In contrast, the detection of infectious virus in the field-collected PQT1 egg pool supports vertical transmission but does not distinguish between transovarial transmission and external contamination of the egg surface. Viral infectivity was confirmed by plaque-forming assay (PFA), and infectious titers were expressed as plaque-forming units per milliliter (PFU/mL), as described in the Section 2 [46,47].

**Figure 3:**
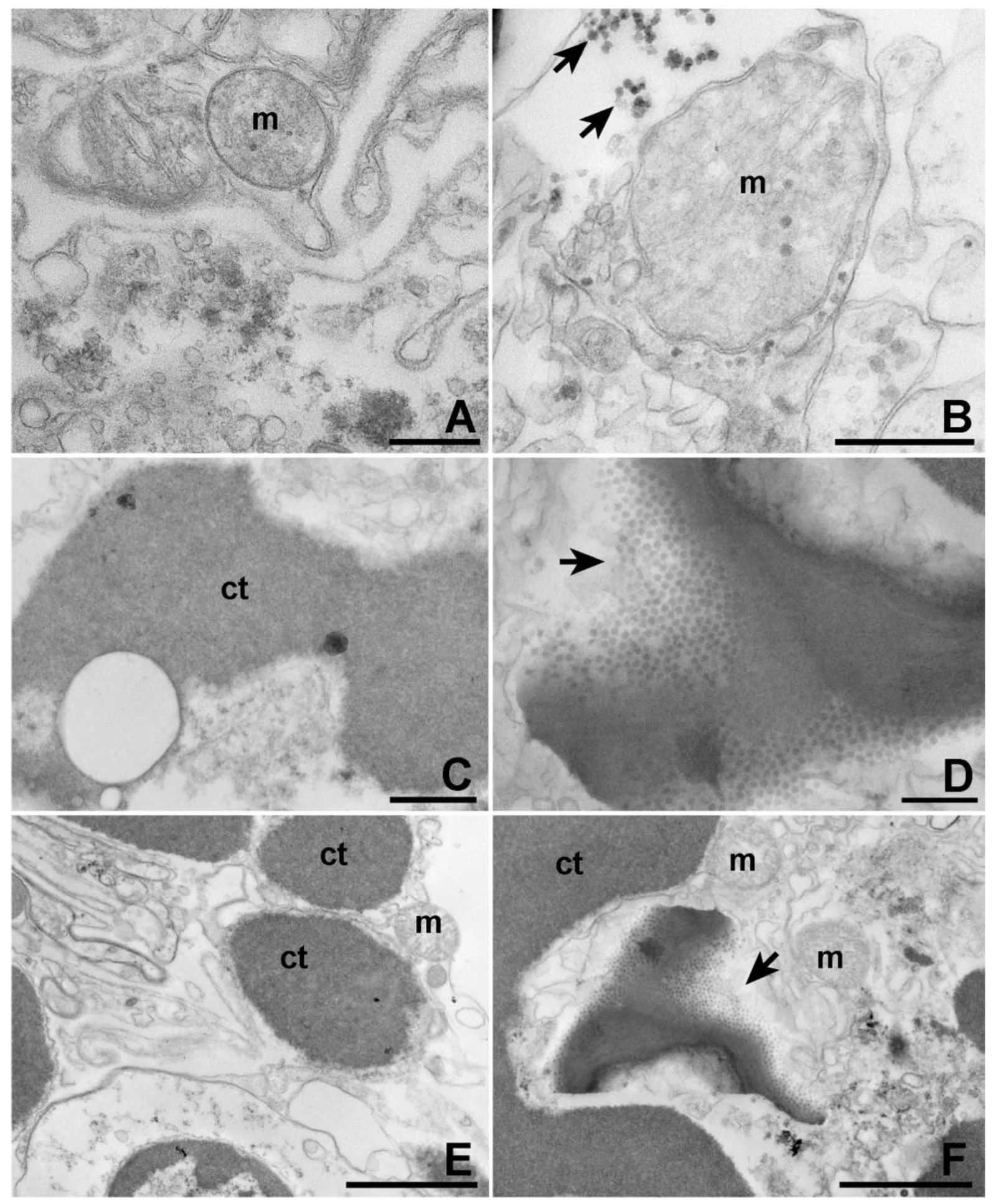
Transmission electron microscopy of infected *Aedes aegypti* eggs. Eggs collected in the South Zone of Rio de Janeiro (Brazil) were macerated and analyzed by transmission electron microscopy. (**A**,**C**,**E**) Rockefeller strain eggs (mock control); (**B**,**D**,**F**) field-collected infected eggs (UR1). (**A**) Mitochondria (m) in a non-infected egg are shown in comparison with a di-lated mitochondrion observed in an infected egg (**B**), which appears to exhibit disrupted membrane integrity. Clusters of viral particles (arrows) are observed surrounding the organelle. Electron-dense material, likely associated with chitin production (ct), is shown in mock (**C**) and infected (**D**) eggs; in the latter, viral particles (arrows) are observed in proximity to this structure. Lower-magnification images of mock (**E**) and infected (**F**) eggs further highlight morphological differences in chitin-associated structures (ct) and mitochondria (m). Viral particles of approximately 50 nm in diameter are indicated by arrows.

Transmission electron microscopy performed on the triturated UR1 DENV-positive eggs revealed spherical virus-like particles approximately 50 nm in diameter within egg tissues (Figure 3). The morphology and size of these particles are consistent with flavivirus virions and complement the molecular, virological, and sequencing findings obtained for this sample. No virus-like particles were observed in the mock control (Rockefeller strain) processed under the same experimental conditions (Figure 3). Collectively, these complementary results provide additional evidence supporting the presence of infectious DENV in naturally collected *A. aegypti* eggs and reinforce the potential of egg-based analysis as a complementary approach for epidemiological surveillance.

## 4. Discussion

In the present study, DENV-1, DENV-2, and DENV-4 were detected in naturally collected *A. aegypti* egg pools using complementary molecular approaches. In addition, infectious virus was recovered from selected egg samples, and virus-like particles consistent with flaviviruses were observed by transmission electron microscopy in egg homogenates. Collectively, these findings provide additional evidence that naturally collected *A. aegypti* eggs can harbor infectious DENV, supporting the occurrence of vertical transmission in natural mosquito populations.

Although DENV vertical transmission has been documented previously, most studies have relied on the detection of viral RNA in larvae, adult mosquitoes, or experimentally infected colonies [27,30,37,48]. In contrast, the present study focused on naturally collected *Aedes aegypti* eggs and combined framework including RT-qPCR, conventional PCR, Sanger sequencing, plaque-forming assays, and transmission electron microscopy to demonstrate not only the presence of viral RNA but also the recovery of infectious virus and the visualization of virus-like particles. Together, these complementary approaches provide robust evidence that naturally collected mosquito eggs may serve as reservoirs of infectious DENV.

The laboratory infection experiment further strengthened these findings. DENV-3 was detected in orally infected female mosquitoes (MA1) and in their corresponding egg pools (EA3), whereas the mock control groups remained negative.

A major novelty of the present study was the detection of DENV in F2 egg samples (E1–E4) of the AEDES-RIO strain, established from field-collected females. Sample E1 was positive for DENV-1, whereas samples E2–E4 were positive for DENV-4, providing evidence consistent with transovarial vertical transmission. Moreover, the E4 egg homogenate successfully infected Vero cells, demonstrating that the virus remained infectious after vertical transmission. Together, these findings indicate that mosquito eggs can harbor infectious DENV and support their potential role in viral persistence, reinforcing the value of egg-based surveillance for monitoring dengue virus circulation in mosquito populations.

The detected serotypes matched those reported to be circulating in Rio de Janeiro by the Brazilian Ministry of Health and national reference laboratories during the same period, supporting the reliability of egg-based detection for DENV surveillance and providing evidence consistent with transovarial vertical transmission [8,49,50].

In subsequent years, eggs collected from Urca (UR1—2015 and UR2—2016), Paquetá Island (PQT—2016), and Bangu/Vila Kennedy (CP1-CP3—2016) consistently tested positive for DENV-1. Notably, co-detection of DENV-1 and DENV-2 was observed in the PQT and CP1–CP3 samples. The co-circulation of multiple serotypes in field-collected egg pools suggests that vertical transmission is not restricted to a single DENV serotype within mosquito populations, but instead reflects the diversity of viruses circulating in the environment. This observation is consistent with epidemiological data documenting the re-emergence and expansion of DENV-2 transmission in Brazil between 2015 and 2016, a period characterized by an accentuated increase in dengue incidence, shifts in serotype predominance, and greater viral diversity [8,51].Together, these findings provide further evidence supporting the natural occurrence of vertical transmission of DENV in *A. aegypti*, consistent with previous reports in mosquitoes of the genus *Aedes* [24,25,38].

Additional laboratory analyses provided complementary evidence for the biological relevance of these observations. The plaque-forming assay (PFA) revealed cell lysis in Vero cells infected with macerated eggs from samples PQT and E4 (Figure 2) that demonstrates the presence of viable infectious viral particles within egg tissues. This is an essential distinction, as indicates that the infectious virus that was recovered in eggs is not merely residual or degraded genetic material but represents replication-competent virus. The ability of viruses to remain infectious and replicate in tissues derived from embryonated eggs is well documented for several arboviruses and other RNA viruses, forming the basis of classical vaccine production platforms, such as those used for influenza and yellow fever [52,53]. In these systems, viruses replicate efficiently in egg-associated tissues, maintaining infectivity and enabling large-scale viral propagation. The recovery of infectious DENV from naturally collected *A. aegypti* egg pools demonstrates that viable viruses can persist in field-collected eggs. Combined with the molecular and ultrastructural analyses, these results provide robust evidence that mosquito eggs can harbor infectious DENV and underscore the value of egg-based analysis as a complementary approach for entomological surveillance. Accordingly, transmission electron microscopy identified virus-like particles in UR1 egg homogenates (Figure 3). These data demonstrate the feasibility of DENV detection in *A. aegypti* eggs, complementary approach for epidemiological surveillance and strengthening public health efforts for the prevention of dengue outbreaks.

The evidence presented here corroborates previous studies reporting evidence of natural vertical transmission of DENV in *Aedes* mosquitoes, including detections in larvae, pupae, and adult males [24,25,27,30,38,54]. Additional field and laboratory investigations have consistently shown DENV in immature stages, confirming vertical transmission as a recurrent biological phenomenon across multiple regions and serotypes [27,32,37,48,54,55].

Our findings extend this knowledge by identifying both single-serotype and multi-serotype DENV-positive egg pools, including co-infections of DENV-1 and DENV-2 in eggs, consistent with prior detections of multiple serotypes in immature forms and adult males in Mexico, Brazil, and the Caribbean [27,30,54,56]. This vertical transmission mechanism has long been considered a potential contributor to viral maintenance during inter-epidemic periods, as vertically infected eggs may generate infected adults even when human transmission is low [14,15,37]. Supporting this hypothesis, infections have been documented during seasons with minimal horizontal transmission [54].

The detection of DENV in field-collected egg pools from different urban settings further supports previous evidence that vertical transmission occurs under natural conditions in geographically distinct *A. aegypti* populations. Studies from Brazil, Mexico, and French Guiana report DENV RNA in male and female mosquitoes and in early larvae, supporting its general occurrence [26,27,30,57,58]. Although previous studies have suggested that egg-based monitoring may complement conventional surveillance strategies, the present study was not designed to evaluate the temporal relationship between DENV detection in eggs and human dengue cases. Therefore, longitudinal studies integrating entomological, virological, and epidemiological data will be required to determine the practical value of this approach for surveillance programs [27,30].

## 5. Conclusions

In conclusion, this study provides additional evidence that naturally collected *A. aegypti* eggs can harbor different DENV serotypes by integrating complementary molecular, virological, and ultrastructural approaches. The combined use of RT-qPCR, conventional PCR, Sanger sequencing, plaque-forming assays, and transmission electron microscopy not only confirmed the presence of viral RNA, but also demonstrated the recovery of infectious virus from egg samples. Moreover, the detection of DENV in F2 eggs derived from field-collected females provides evidence consistent with transovarial vertical transmission. Although this study was not designed to distinguish transovarial transmission from transovum contamination in field-collected eggs or to evaluate the relationship between DENV detection in eggs and subsequent dengue outbreaks, the findings highlight the value of egg-based analysis as a complementary tool for entomological surveillance. Future longitudinal studies integrating vector surveillance, complete viral genome sequencing, and epidemiological data will be essential to clarify the contribution of vertically infected eggs to DENV persistence during interepidemic periods and to assess their potential application in dengue surveillance programs.

## Figures and Tables

**Table 1:** Summary of Egg Sample Collections and Dengue Virus Detection.

Sample	Location	Collection Year	DENV1	DENV-2	DENV-3	DENV-4
E1	F2 eggs of the AEDES-RIO strain *	2013–2014	+	−	−	−
E2	F2 eggs of the AEDES-RIO strain *	2013–2014	−	−	−	+
E3	F2 eggs of the AEDES-RIO strain *	2013–2014	−	−	−	+
E4	F2 eggs of the AEDES-RIO strain *	2013–2014	−	−	−	+
UR1	Urca	2015	+	−	−	−
UR2	Urca	2016	+	−	−	−
UR3	Urca	2015	−	−	−	−
UR4	Urca	2016	−	−	−	−
PQT-1	Paquetá Island	2016	+	+	−	−
PQT-2	Paquetá Island	2016	−	−	−	−
CP1	Bangu	2016	+	+	−	−
CP2	Vila Kennedy	2016	+	+	−	−
CP3	Vila Kennedy	2016	+	+	−	−

* Egg data obtained from the work of Santos et al., [45].

**Table 2:** Detection of DENV in field-collected *Aedes aegypti* egg pools from Rio de Janeiro, Brazil.

Collection Site	Egg Pools Analyzed (n)	Positive Pools (n)	Eggs Analyzed (n)	Detection Frequency (%)	DENV Serotype(s) Detected
E1	1	1	300	100	DENV-1
E2	1	1	300	100	DENV-4
E3	1	1	300	100	DENV-4
E4	1	1	300	100	DENV-4
UR1	1	1	300	100	DENV-1
UR2	1	1	300	100	DENV-1
UR3	1	0	300	0	ND
UR4	1	0	300	0	ND
PQT	2	1	250	50	DENV-1/DENV-2
CP	3	3	400	100	DENV-1/DENV-2
Total	13	10	3050	76.9	DENV-1, DENV-2 and DENV-4

## Data Availability

The original contributions presented in this study are included in the article/Supplementary Material. Further inquiries can be directed to the corresponding author.

## Supplementary Materials

The following supporting information can be downloaded at https://www.mdpi.com/article/10.3390/microorganisms14081810/s1. File S1: Conventional Nested PCR for Sanger Sequencing. Figure S1: Geographic location of the *Aedes aegypti* egg collection sites included in this study. Egg samples were collected from Ilha do Governador (green), Ilha de Paquetá (orange), Vila Kennedy (yellow), Bangu (red), Urca (cyan), São Gonçalo (purple), and Niterói (blue), located in the City of Rio de Janeiro and the Metropolitan Region, Brazil; Table S1: Mean Ct values and estimated viral copy numbers obtained by serotype-specific RT-qPCR for laboratory and field-collected *Aedes aegypti* egg samples; Table S2: RT-qPCR results obtained from experimentally infected *Aedes aegypti* females and their corresponding egg pools.

## Abbreviations

The following abbreviations are used in this manuscript:

*A. aegypti*	*Aedes aegypti*
ARBOV	Arbovirus
BSL-2	Biosafety Level 2
C6/36	*Aedes albopictus* cell line
cDNA	Complementary DNA
CMC	Carboxymethylcellulose
${\mathrm{CO}}_{2}$	Carbon dioxide
Ct	Chitin-associated structure
DENV	Dengue virus
DENV-1	Dengue virus serotype 1
DENV-2	Dengue virus serotype 2
DENV-3	Dengue virus serotype 3
DENV-4	Dengue virus serotype 4
DHF	Dengue hemorrhagic fever
DMEM	Dulbecco’s Modified Eagle Medium
DMP-30	2,4,6-tris(dimethylaminomethyl)phenol
DNA	Deoxyribonucleic acid
DSS	Dengue shock syndrome
EA3	Egg sample group 3
E1–E4	Egg sample groups 1–4
FBS	Fetal bovine serum
ICTV	International Committee on Taxonomy of Viruses
LMTH	Laboratório de Mosquitos Transmissores de Hematozoários
MA1	Mosquito adult sample 1
m	Mitochondria
MOI	Multiplicity of infection
NCBI	National Center for Biotechnology Information
NIH	National Institutes of Health
${\mathrm{OsO}}_{4}$	Osmium tetroxide
PFA	Plaque Forming Assay
PFU	Plaque Forming Units
PBS	Phosphate-buffered saline
PDTIS	Programa de Desenvolvimento Tecnológico em Insumos para Saúde
PQT	Paquetá sample group
qPCR	Quantitative polymerase chain reaction
RNA	Ribonucleic acid
RT-qPCR	Reverse transcription quantitative polymerase chain reaction
SYBR	SYBR Green fluorescent dye
Tm	Melting temperature
UR1/UR2	Urca sample groups
Vero cells	African green monkey kidney epithelial cells
YFV	Yellow fever virus
ZIKV	Zika virus
