# Supplementary material for: Detection of Dengue Virus in Aedes aegypti Eggs Supports Vertical Transmission in Natural Settings
Source: Microorganisms. 2026 Aug 17;14(8):1810. doi: 10.3390/microorganisms14081810 (PMC13515839; doi:10.3390/microorganisms14081810)
Supplement: Supplementary file 1 [file microorganisms-14-01810-s001.zip › Figure Supplementary S1.pdf]

Detection of Dengue Virus in *Aedes aegypti* Eggs Supports Vertical Transmission in Natural Settings

Tiago S. Salles, Brenda M. Vasconcellos, Carlucio R dos Santos, Maria Luiza Maia, Svetoslav Nanev Slavov, Renata C. Avezedo, Lúcio A. Caldas and Monica F. Moreira

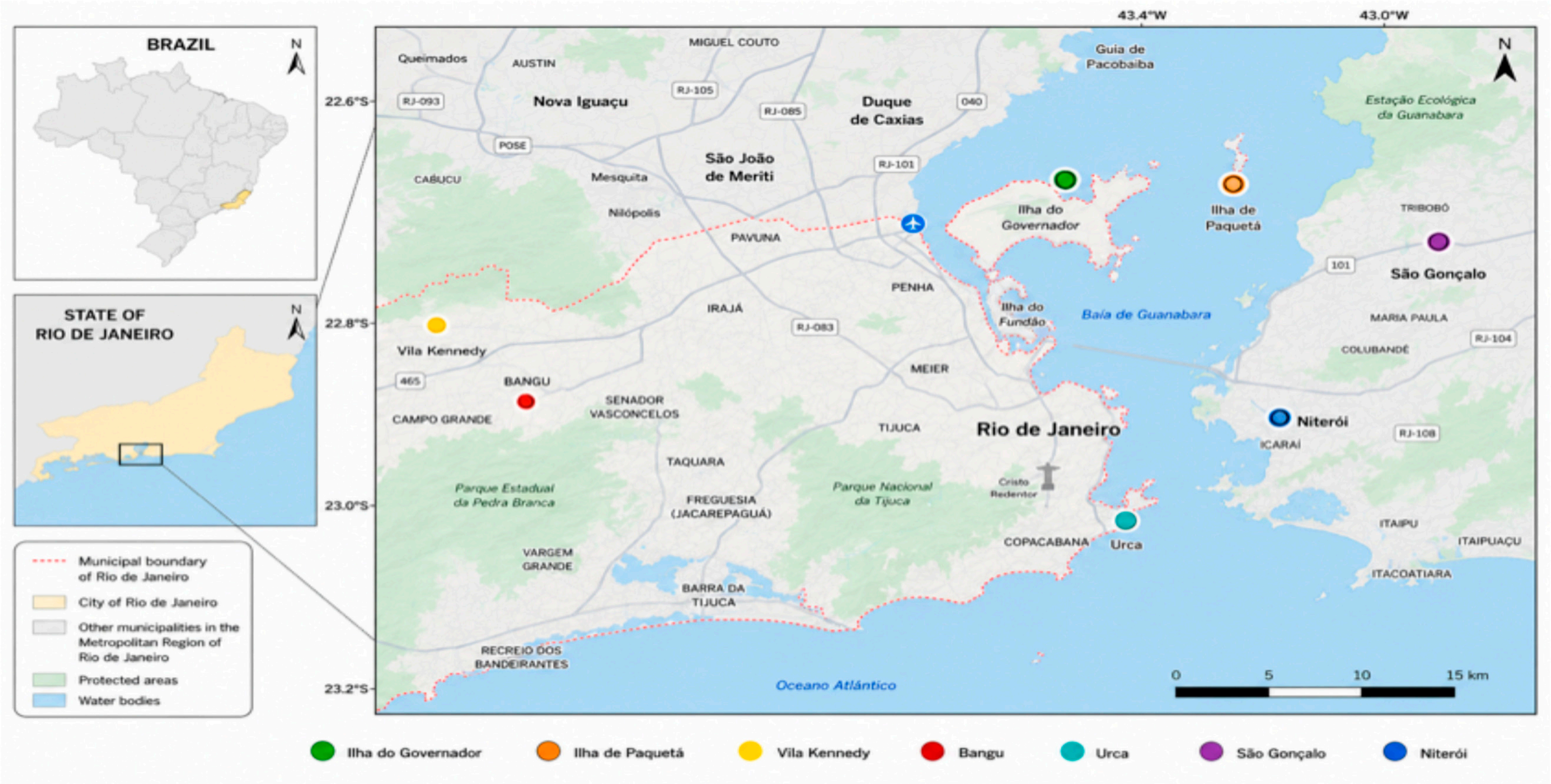

Figure Supplementary S1. Geographic location of the *Aedes aegypti* egg collection sites included in this study. Egg samples were collected from Ilha do Governador (green), Ilha de Paquetá (orange), Vila Kennedy (yellow), Bangu (red), Urca (cyan), São Gonçalo (purple), and Niterói (blue), located in the City of Rio de Janeiro and the Metropolitan Region, Brazil.
