# Supplementary material for: Detection of Dengue Virus in Aedes aegypti Eggs Supports Vertical Transmission in Natural Settings
Source: Microorganisms. 2026 Aug 17;14(8):1810. doi: 10.3390/microorganisms14081810 (PMC13515839; doi:10.3390/microorganisms14081810)
Supplement: Supplementary file 1 [file microorganisms-14-01810-s001.zip › Supplementary File S1.pdf]

Detection of Dengue Virus in *Aedes aegypti* Eggs Supports Vertical Transmission in Natural Settings

Tiago S. Salles, Brenda M. Vasconcellos, Carlucio R dos Santos, Maria Luiza Maia, Svetoslav Nanev Slavov, Renata C. Avezedo, Lúcio A. Caldas and Monica F. Moreira

Supplementary Material

Conventional Nested PCR for Sanger Sequencing

To confirm the DENV-positive samples identified by RT-qPCR, a nested conventional PCR was performed as previously described by Salles et al. (2017). Briefly, the first-round PCR consisted of a 10 μL reaction containing 1 μL of cDNA template, 100 nM of the universal DENV primers (STD and D2), and Phusion High-Fidelity DNA Polymerase reagents (Thermo Fisher Scientific, Waltham, MA, USA), following the manufacturer's instructions.

The second-round PCR was carried out in a final volume of 20 μL containing 1 μL of the first-round amplification product, Phusion High-Fidelity DNA Polymerase with HF buffer (Thermo Fisher Scientific), and 50 nM of each serotype-specific primer pair (STD1/rTS1, STD2/mTS2, STD3/TS3, and STD4/rTS4), allowing discrimination of DENV-1 to DENV-4.

Amplification products were analyzed by electrophoresis on a 1.5% agarose gel, and DENV serotypes were initially identified according to the expected amplicon size described by Salles et al. (2017). PCR products obtained from positive samples were subsequently purified and subjected to Sanger sequencing to confirm the identity of the detected DENV serotypes. The resulting sequences were analyzed by comparison with reference DENV sequences available in GenBank using the BLASTn algorithm.

Sample E4

GAATAGAGAGACGCGTATCACCCCTCAGGGTTGGTGAAGAGATTCTCAACC
GGACTTTTTTCTGGGAAAGGACCCTTACGGATGGTGCTAGCATTCATCACG
TTTTTGCGAGTCCTTTCCATCCCACCAACAGCAGGGATTCTGAAGAGATGG
GGACAGTTGAAGAAAAATAAGGCCATCAAGATACTGATTGGATTCAGGAAG
GAGATAGG

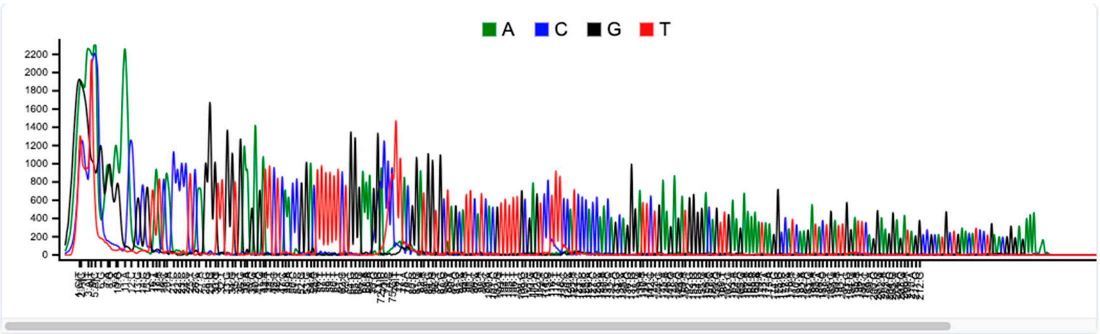

Extracted Sequence

View Full

GAATAGAGAGACGCGTATCACCCCTCAGGGTTGGTGAAGAGATTCTCAACCGGACTTTTTTCTGGGAAAGGACCCTTACGGATGGTG
CTAGCATTCATCACGTTTTTGCGAGTCCTTTCCATCCCACCAACAGCAGGGATTCTGAAGAGATGGGGACAGTTGAAGAAAAATAAG
GCCATCAAGATACTGATTGGATTCAGGAAGGAGATAGG

Sample PQT

CGTCTTGCAGCATTCCAGTGAGAATCTCTTTGTCAGCTGTTGCACAGTTGA
CACGCGGTTTCTCTCGCGTTTCAGCATATTGAAAGGCGTGTTTTTCGCAAT
GTGCCCCTTAAA

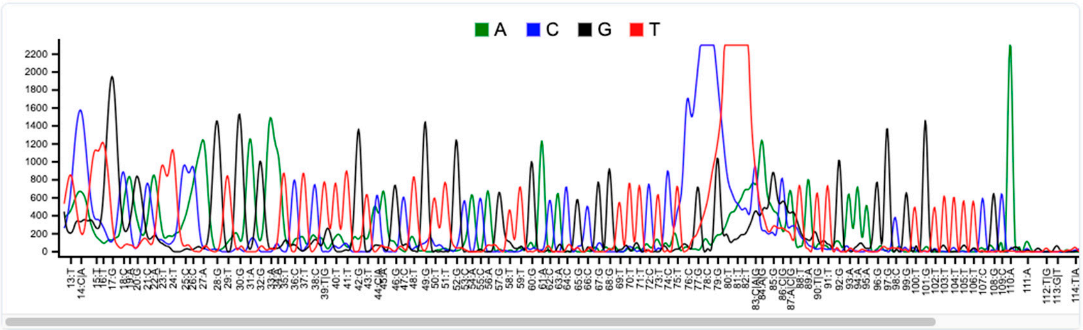

Extracted Sequence

View Full

ACAAATGTCTCG**TCTTGCAGCATTCCAGTGAGAATCTCTTTGTCAGCTGTTGCACAGTTGACACGCGGTTTCTCTCGCGTTTCAGCA**
TATTGAAAGGCGTGTTTTTCGCAATGTGCCCCTTAAANGGCCTAAGGTTTTTTCCAGAAAAAAGTCCGGNAGAGAATCTCGTCCCCA
CAGAGGAGNGTTTATTCCTCTANNNGNCG

Sample CP1

ACGGACTTTGATGAGCTCACCATAGCCAAATTCCTGCTGTTGGGGGTATGG
CATAGAAAGTGCTGTAGAATATGCTGATGAAAGGCATCACCAATTTCATGGG
GCCTTGGCCTGAAAGCAATCCTTTTGAGAATCTCTTCGCCAACTGTGAACC
AGTTGACACGCGGTTTCTCGCGCGTTTCAGCATATTGAAAGACGGTCGACC
CGTTTATTTTCTCTCGCGTTTCAGCATATTGAAAGGTGGTCAAACCACAAAA
TTGGGAAGGGGACTTACTCGAAATACCTGGATC

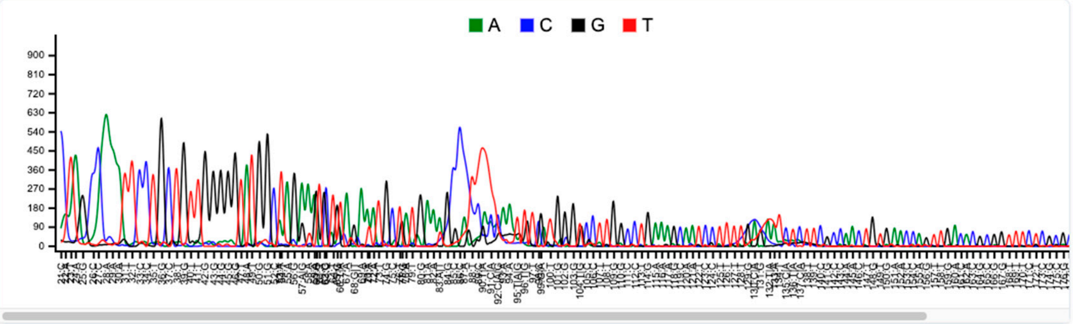

Extracted Sequence

View Full

ACGAACTTTGATGAGCTCAC**CATAGCCAAATTCCTGCTGTTGGGGGTATGGCATAGAAAGTGCTGTAGAATATGCTGATGAAAGCCA**
TCTCCAATTTCGTGGGTCCTTGGCCTGAAAGCAATCCTTTTGCGTATCTCTTCGCCAACTGTGAACCAGTTGACACGCGGTTTCTCG
CGCGTTTCAGCATATTGAAAGACGGTCGACCCGTATANATTNTCTCGCGTTTCAGCATATTGAAAGGTGGTGTAACCACAGAATNGN
CAANGNNNGTNGNNNGANANANNAGGNTN
