# Supplementary material for: Detection of Dengue Virus in Aedes aegypti Eggs Supports Vertical Transmission in Natural Settings
Source: Microorganisms. 2026 Aug 17;14(8):1810. doi: 10.3390/microorganisms14081810 (PMC13515839; doi:10.3390/microorganisms14081810)
Supplement: Supplementary file 1 [file microorganisms-14-01810-s001.zip › Supplementary Table S1.pdf]

Detection of Dengue Virus in *Aedes aegypti* Eggs Supports Vertical Transmission in Natural Settings

Tiago S. Salles, Brenda M. Vasconcellos, Carlucio R dos Santos, Maria Luiza Maia, Svetoslav Nanev Slavov, Renata C. Avezedo, Lúcio A. Caldas and Monica F. Moreira

Supplementary Table S1. Mean Ct values and estimated viral copy numbers obtained by serotype-specific RT-qPCR for laboratory and field-collected *Aedes aegypti* egg samples.

Sample	DENV-1 Mean Ct	DENV-1 Copy Number	DENV-2 Mean Ct	DENV-2 Copy Number	DENV-3 Mean Ct	DENV-3 Copy Number	DENV-4 Mean Ct	DENV-4 Copy Number
LA-1	N/A	N/A	N/A	N/A	34.10	7.15×10^2	N/A	N/A
LA-2	N/A	N/A	N/A	N/A	N/A	N/A	N/A	N/A
LA-3	N/A	N/A	N/A	N/A	33.44	6.98×10^2	N/A	N/A
LA-4	N/A	N/A	N/A	N/A	N/A	N/A	N/A	N/A
E1	34.05	7.93×10^0	N/A	N/A	N/A	N/A	N/A	N/A
E2	N/A	N/A	N/A	N/A	N/A	N/A	N/A	N/A
E3	34.71	8.13×10^0	N/A	N/A	N/A	N/A	35.40	8.09×10^1
E4	N/A	N/A	N/A	N/A	N/A	N/A	36.43	8.40×10^0
UR1	34.54	8.08×10^0	N/A	N/A	N/A	N/A	N/A	N/A

UR2	33.79	7.85×10^1	N/A	N/A	N/A	N/A	N/A	N/A
UR3	N/A	N/A	N/A	N/A	N/A	N/A	N/A	N/A
UR4	N/A	N/A	N/A	N/A	N/A	N/A	N/A	N/A
PQT1	30.45	6.85×10^2	30.31	7.58×10^2	N/A	N/A	N/A	N/A
PQT2	N/A	N/A	N/A	N/A	N/A	N/A	N/A	N/A
CP1	29.84	6.67×10^2	34.07	8.76×10^0	N/A	N/A	N/A	N/A
CP2	32.70	7.52×10^1	34.83	9.00×10^0	N/A	N/A	N/A	N/A
CP3	31.00	7.01×10^1	33.72	8.65×10^1	N/A	N/A	N/A	N/A
PLD 10	8.87	3.60×10^9	8.25	6.70×10^8	11.61	1.13×10^8	14.67	1.26×10^8
PLD 10^2	13.28	1.69×10^8	12.21	1.91×10^8	15.69	2.22×10^6	17.82	2.42×10^7
PLD 10^3	15.98	2.50×10^7	14.01	2.47×10^7	19.30	3.19×10^5	20.91	3.39×10^6
PLD 10^4	21.06	4.03×10^5	21.46	4.81×10^5	23.26	4.25×10^4	24.64	2.42×10^5
PLD 10^5	23.71	4.82×10^4	24.85	5.87×10^4	29.12	5.82×10^3	27.72	5.06×10^4
PLD 10^6	27.45	5.95×10^3	28.19	6.92×10^3	33.28	2.42×10^2	31.80	2.42×10^3
cDNA	26.11	5.54×10^3	33.70	8.64×10^1	27.72	5.44×10^3	22.06	4.15×10^4
Negative control	N/A	N/A	N/A	N/A	N/A	N/A	N/A	N/A
