# Supplementary material for: Detection of Dengue Virus in Aedes aegypti Eggs Supports Vertical Transmission in Natural Settings
Source: Microorganisms. 2026 Aug 17;14(8):1810. doi: 10.3390/microorganisms14081810 (PMC13515839; doi:10.3390/microorganisms14081810)
Supplement: Supplementary file 1 [file microorganisms-14-01810-s001.zip › Supplementary Table S2.pdf]

Detection of Dengue Virus in *Aedes aegypti* Eggs Supports Vertical Transmission in Natural Settings

Tiago S. Salles, Brenda M. Vasconcellos, Carlucio R dos Santos, Maria Luiza Maia, Svetoslav Nanev Slavov, Renata C. Avezedo, Lúcio A. Caldas and Monica F. Moreira

Supplementary Table S2. RT-qPCR results obtained from experimentally infected *Aedes aegypti* females and their corresponding egg pools.

Experimental group	Pool	Interpretation	Ct value
MA1 (infected females)	Pool 1	Positive	33.35
MA1 (infected females)	Pool 2	Positive	33.43
MA1 (infected females)	Pool 3	Positive	33.55
EA3 (eggs from infected females)	Pool 1	Positive	33.47
EA3 (eggs from infected females)	Pool 2	Positive	34.00
EA3 (eggs from infected females)	Pool 3	Positive	34.81
MA2 (mock females)	Pool 1	Negative	ND
MA2 (mock females)	Pool 2	Negative	ND
MA2 (mock females)	Pool 3	Negative	ND
EA4 (eggs from mock females)	Pool 1	Negative	ND
EA4 (eggs from mock females)	Pool 2	Negative	ND
EA4 (eggs from mock females)	Pool 3	Negative	ND

ND, not detected. MA1: experimentally infected female mosquito pools; MA2: mock-infected female pools; EA3: egg pools obtained from experimentally infected females; EA4: egg pools obtained from mock-infected females.
